# *Internal transcribed spacer* as effective molecular marker for the detection of natural hybridization between the bivalves *Pinna nobilis* and *Pinna rudis*

**DOI:** 10.1002/ece3.70227

**Published:** 2024-08-26

**Authors:** Gaetano Catanese, Maite Vázquez‐Luis, Salvatore Giacobbe, José Rafael García‐March, Maria Zotou, Prado Patricia, Orestis Papadakis, José Tena‐Medialdea, Stelios Katsanevakis, Amalia Grau

**Affiliations:** ^1^ IRFAP LIMIA (Laboratorio de Investigaciones Marinas y Acuicultura) – Govern de les Illes Balears Port d'Andratx Balearic Islands Spain; ^2^ IEO‐CSIC, Centro Oceanográfico de Baleares Instituto Español de Oceanografía Palma de Mallorca Spain; ^3^ Department of Chemical, Biological, Pharmaceutical and Environmental Sciences, ChiBioFarAm Università Degli Studi di Messina Messina Italy; ^4^ Instituto de Investigación en Medio Ambiente y Ciencia Marina (IMEDMAR‐UCV) Universidad Católica de Valencia Calpe Spain; ^5^ Department of Marine Sciences University of the Aegean Mytilene Greece; ^6^ Institut d'Estudis Professionals Aqüícoles i Ambientals de Catalunya (IEPAAC) Tarragona Spain; ^7^ IRTA‐La Ràpita Tarragona Spain

**Keywords:** DNA barcode, endangered species, hybrid detection, molecular tools, multiplex PCR, ribosomal unit

## Abstract

The *Pinna nobilis*, a Mediterranean mollusc, has suffered population declines due to a massive mortality event associated with various factors including the parasite *Haplosporidium pinnae*. Some populations show resilience, possibly due to local environmental conditions. In this study, a molecular multiplex PCR method was developed using species‐specific primers targeting Internal Transcribed Spacer (ITS) regions of *P. nobilis* and *P. rudis*, allowing accurate species identification and hybrid detection. Samples from Mediterranean areas were analysed, including putative hybrids and individuals from five other bivalve species. DNA was isolated, ITS regions were amplified and sequenced, and phylogenetic analyses confirmed species differentiation and primer specificity. The multiplex‐PCR successfully identified *P. nobilis*, *P. rudis*, and their hybrids based on distinct amplicon patterns. This study highlights the value of molecular tools in species conservation, especially for monitoring and managing hybridization, supporting effective biodiversity conservation strategies.

## Graphical abstract

The Pinna nobilis, a Mediterranean mollusc, has suffered population declines due to a mortality event linked to the parasite Haplosporidium pinnae, though some populations show resilience. This study developed a molecular multiplex‐PCR method using species‐specific primers to accurately identify *P. nobilis*, *P. rudis*, and their hybrids. The method's effectiveness was confirmed through analysis of samples from various Mediterranean areas, highlighting its value for species conservation and management.

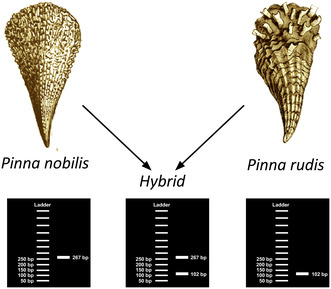

## 1 INTRODUCTION

In recent years, the Mediterranean endemic bivalve *Pinna nobilis*, commonly known as the noble pen shell or fan mussel, has undergone a massive mortality event (MME). It has manifested as a rapid and widespread mortality of individuals throughout the Mediterranean Sea, raising concerns about the species' survival (Katsanevakis et al., 2022; Kersting et al., 2019). The exact cause of the MME in the Mediterranean Sea is still debated, and different hypotheses have been proposed to explain its origin. Multifactorial causes involving a combination of factors such as pathogens, parasites or environmental stressors have been also suggested (Box et al., 2020; Carella et al., 2019; Cižmek et al., 2020; Lattos et al., 2020; Scarpa et al., 2020). Nevertheless, one of the main pathogens suspected to be involved in the decline of *Pinna nobilis* populations is the protozoan parasite, *Haplosporidium pinnae* (Catanese et al., 2018; Darriba, 2017), which has been found in affected individuals and whose infection has been shown to play a major role in the onset of the mass mortality (Grau et al., 2022; Tiscar et al., 2022). More recently, an RNA virus has been also associated with disease and mortality in this species due to the virus's long‐term weakening effect on animals, increasing susceptibility and rendering them more vulnerable to pathogenic parasite attacks (Carella et al., 2023). Unfortunately, *H. pinnae* is now spreading even to previously presumed sanctuaries, areas that had remained *H. pinnae*‐free due to natural barriers such as river mouths, gulfs, and coastal marine lagoons (Donato et al., 2021, 2023; Foulquié et al., 2023; Labidi et al., 2023; Moro‐Martínez et al., 2023; Nebot‐Colomer et al., 2021; Prado et al., 2021). In some instances, summer water restrictions, like those observed in 2023 at the Delta Ebro region (Spain), may have disrupted the natural protective barrier of salinity gradients, potentially leading to an increased rate of parasite infections (CHEbro, 2023). In any case, *H. pinnae* seems to exhibit high species specificity, infecting solely *P. nobilis* and not its congeneric species *Pinna rudis* (Catanese et al., 2018; Vázquez‐Luis et al., 2017). Some populations of *P. nobilis* have currently remained alive apparently thanks to specific local abiotic conditions such as reduced temperature and extreme salinity (below or above typical Mediterranean Sea salinity), which hinder the spread of the pathogens (Acarlı et al., 2024; Cabanellas‐Reboredo et al., 2019; Çınar et al., 2021; Cortés‐Melendreras et al., 2022; Giménez‐Casalduero et al., 2020; Prado et al., 2021) or result in a greater host resilience to infections (Coupé et al., 2023; Karadurmuş et al., 2024; Papadakis et al., 2023; Salis et al., 2022). Such is the case of *P. nobilis* populations in the Sea of Marmara, where there is no documentation of pathogen infection, attributed to the low salinity of the region (Acarli et al., 2022; Karadurmuş et al., 2024). These populations can act as source of repopulation through larval dispersal, which could be transported by sea currents over large distances to other regions by sea currents, even to hundreds of kilometres away (Feria‐Rodríguez et al., 2024; Katsanevakis, 2016; Kersting et al., 2020; Kersting & García‐March, 2017; Papadakis et al., 2023; Peyran et al., 2022).

Scientists, environmentalists and government organizations are collaborating to implement a strategy for monitoring and safeguarding the species and to apply conservation and repopulation measures for surviving populations (García‐March et al., 2020). For instance, efforts to monitor reproductive events in the wild for juvenile recruitment have led to the deployment of collectors in various regions of the Mediterranean Sea (Cabanellas‐Reboredo et al., 2009; Kersting et al., 2020; Kersting & García‐March, 2017; Nebot‐Colomer et al., 2024). Through these methods, captured juveniles can also be bred under long‐term captivity conditions (Cortés‐Melendreras et al., 2022; García‐March et al., 2020; Hernandis et al., 2022, 2023; Prado et al., 2020) screened for pathogens, and then transferred to areas for repopulation initiatives of affected populations (Katsanevakis, 2016; Kersting et al., 2020; Kersting & García‐March, 2017).

Identifying juveniles of *Pinna* spp. based on specific shell features is feasible due to their small size, but distinguishing between different *Pinna* species can be challenging (Catanese, Tena‐Medialdea, et al., 2022). The congeneric species *P. rudis* is occasionally found in the Mediterranean, and it can be mainly found in waters from the Caribbean to the eastern Atlantic Ocean (Gvozdenović Nikolić et al., 2019; Huber, 2010). Recently it appears to have shown significant expansion into the Mediterranean Sea, as observed within Greek waters and documented through dedicated surveys and molecular confirmation. This expansion indicates an ecological phenomenon whereby *P. rudis* appears to be colonizing new habitats within the Mediterranean basin, some of which were previously occupied by *P. nobilis* (Kersting & Ballesteros, 2021; Oprandi et al., 2024; Zotou et al., 2023). During the diving surveys, some researcher observed individuals exhibiting morphological traits that seemed to be a mixture of the two *Pinna* species (Rubino et al., 2024; Vázquez‐Luis et al., 2021). Therefore, species sharing the same area may come into contact due to changes in habitat or environmental conditions, creating opportunities for hybridization. In fact, without spatial isolation, closely related species tend to hybridize more often (Abbott et al., 2013). Hybridization involves a complex interplay of ecological consequences. It can contribute to speciation by creating new hybrid taxa, and introgression of specific genetic loci may promote adaptive divergence, thus facilitating speciation (Abbott et al., 2013). Furthermore, hybridization can lead to the erosion of native genotypes and increase the invasive potential of certain species, enabling their successful establishment and expansion into new habitats (Seehausen, 2013). Hybridization can still occur in the absence of reproductive barriers between species, although it can be harmful and lead to non‐viable offspring (Abbott et al., 2013; Sedghifar et al., 2016).

In nature, the occurrence of natural hybridization among molluscs is quite common and it has been already described in many species including oysters (Huvet et al., 2004) and mussels (Beaumont et al., 2004; Rawson et al., 1996; Voroshilova et al., 2010).

Although rapid identification methods between *P. nobilis* and *P. rudis* have already been described, they are based on the sequencing or multiplex‐PCR amplification of mitochondrial DNA (mtDNA) fragments, which is normally transmitted through maternal inheritance (Catanese, Coupé, & Bunet, 2022; Catanese, Tena‐Medialdea, et al., 2022; Papadakis et al., 2023). Therefore, to identify juvenile or adult hybrids it is necessary to use additional genetic information present on the chromosomes from both parents. Each individual inherits half of its chromosomes from each parent during the breeding process and as a result, mtDNA versus nDNA alone cannot provide sufficiently detailed information to distinguish hybrids from pure lineage parental species.

Recently, morphological characteristics and molecular analyses were used to identify putative hybrids between congeneric *P. nobilis* and *P. rudis* (Vázquez‐Luis et al., 2021). However, employing a molecular method that relies on sequencing three fragments of the 28SrDNA gene to detect particular nucleotide positions, characterized by the presence of two distinct peaks of signal intensity in diagnostic sites, seems comfortless, costly, and susceptible to errors of interpretation.

Within eukaryotes, the Internal Transcribed Spacer (ITS) regions constitute non‐coding stretches situated within the ribosomal DNA repeats amidst the small subunit (18S rDNA) and large subunit (28S rDNA) ribosomal genes. They are subdivided into two primary segments: ITS1 and ITS2 with 5.8S rDNA genes intercalated between them. Both ITSs are used in characterizing eukaryotic organisms at the molecular level to discern species, evaluate biodiversity, and probe evolutionary relationships. Among species, they exhibit divergent lengths, sequence variabilities, and evolutionary dynamics, with applications in multiple domains including ecology, conservation biology, and forensics. The high sequence variation inherent in ITS regions can facilitate the discrimination of closely related taxa, using them as DNA barcodes in molecular biology and taxonomy for marine species identification (Aranishi, 2005; Moniz & Kaczmarska, 2010; Prasad et al., 2009; Schoch et al., 2012; Stern et al., 2012; Wiemers et al., 2009). Moreover, despite the substantial variability in these regions, specific segments flanking or included within them remain conserved across taxa. This conservation allows for the design of universal primers suitable for PCR amplification. The ITS1 region, known for its high variability and preferred use as a DNA barcode in molecular taxonomy, provides enhanced discriminatory power for closely related species, whereas ITS2, although shorter and more conserved than ITS1, features lower sequence variability but retains informative variations crucial for taxonomic and phylogenetic analyses, prompting researchers to often combine ITS1 and ITS2 sequences for comprehensive taxonomic and phylogenetic investigations. The rapid evolutionary rate of the ITS region significantly improves the accurate differentiation of closely related species, serving as an effective tool for plant species identification and as a supplementary marker alongside barcode for animal identification (Yao et al., 2010).

In this work, we developed a molecular multiplex‐PCR procedure using species‐specific primers targeting the ITS regions of *P. nobilis* and *P. rudis*. This approach enables the identification of these species based on the positive amplification of PCR fragments with distinct sizes. Additionally, our method allows for the detection of hybrids between these two species by observing positive amplification of two different PCR fragments.

## 2 MATERIALS AND METHODS

### 2.1 Sampling

A total of 41 biopsies (25 of *P. nobilis* and 16 of *P. rudis*) were collected between 2021 and 2023 in different areas of the Mediterranean Sea, the ratio *P. nobilis*/*P. rudis* in each location was as follows: Delta Ebro (5/0), Calpe (5/4), Balearic Islands (4/1) [Spain]; Messina (5/3) [Italy]; Crete (0/8) and Lesvos Island (6/0) [Greece]. Three putative hybrids were collected from the Balearic Islands (Spain). The biopsies consisted of a portion of the mantle of each individual that was excised and kept in absolute ethanol. All analysed *Pinna* individuals were adults. In addition, we also collected two samples of each species of the following molluscs: *Pinctada* sp., *Spondylus gaederopus*, *Malleus* sp., and *Isognomon* sp. from Messina (Italy) and *Atrina pectinata* from Calpe (Spain). The identification of all these species was mainly based on morphological features. Samplings were performed with the necessary permits of the competent authorities (national and/or local).

### 2.2 DNA isolation, amplification and sequencing

Total genomic DNA was isolated from 30 mg of tissue using the Tissue Genomic DNA Extraction Kit (Macherey‐Nagel), according to the manufacturer's instructions.

For the amplification of the ITS regions, primer pairs Pinna_ITSF (5’‐CCGTTGAACCTCCTTCGTGCT‐3′) and Pinna_ITSR (5’‐GGCTCTTCCCGCTTCACTC‐3′) were designed based on the partial sequences of 18S rDNA and 28S rDNA of *Pinna* available on GenBank. These primers were used in all the bivalve considered in this study, except for PCR amplification of *Atrina* species for which the primers ITS5 and ITS4, designed by White et al. (1990), were applied.

All PCR reactions were performed in a total volume of 20 μL containing: 10 μL of Kapa Taq Ready mix (Sigma‐Aldrich), 8.2 μL of sterile water, 0.4 μL of each primer (stock 20 Mmol) and 1 μL of DNA at 50 ng/μL. The following conditions of PCR were used: an initial denaturation step at 95°C for 3 min, followed by 35 cycles of denaturation at 95°C for 30 s, annealing at 50–60°C for 30 s and elongation at 72°C for 5 min. The PCR products were purified using a mi‐Gel Extraction Kit (Metabion) and bidirectionally sequenced using the Sanger's method at Secugen service (www.secugen.es; 15.02.2024). Species identification of *Pinna* sp. individuals based on mitochondrial DNA pattern was also carried out using the method described by Catanese, Tena‐Medialdea, et al. (2022).

### 2.3 Sequence comparison, genetic distance and phylogenetic analyses

Although, morphologically, the adult individuals of different bivalve families are possibly recognizable, we analysed the sequences of other species to be sure not to design primers that could give cross‐amplification. Therefore, to develop a multiplex PCR assay specific for *P. nobilis* and *P. rudis* identification a profound analysis of the nucleotide differences between the sequences of the considered species was carried out. The nucleotide sequences of ITS regions were edited and aligned using BioEdit (Hall, 1999) and MEGA 11 software (Tamura et al., 2021). The base composition, nucleotide differences and pairwise genetic distances were obtained using MEGA11.

The sequences were then analysed with JModelTest v2.1.7 (Posada, 2008) using the Akaike Information Criterion (AIC; Posada & Buckley, 2004) to select the appropriate model of evolution, as a criterion to determine the best model. A neighbour‐joining (NJ), from Tamura‐Nei genetic distances and a Maximum likelihood (ML) method was adopted to reconstruct the phylogenetic relationships using MEGA11, with 1000 bootstrap replicates. In addition to the obtained sequences for *Pinna* spp. and other molluscs, the following bivalve species ITS sequences described by other authors and retrieved from GenBank were used for sequence comparison: *P. nobilis* (KX101235), *Atrina pectinata* (NC020028), *Spondylus gaederopus* (KX257353) *Mytilus edulis* (AY695798), *M. galloprovincialis* (JX081670), and different species of the *Haliotis* family (from AF296851 to AF296869). The sequence of *Ruditapes decussatus* (HQ634139) was also included in the analysis as an outgroup.

### 2.4 Development of multiplex PCR assay for species identification and hybrid detection

For the development of the assay procedure for detection of hybrid between *P. nobilis* and *P. rudis*, amplicons with different size were considered in the primer design.

**FIGURE 1:**
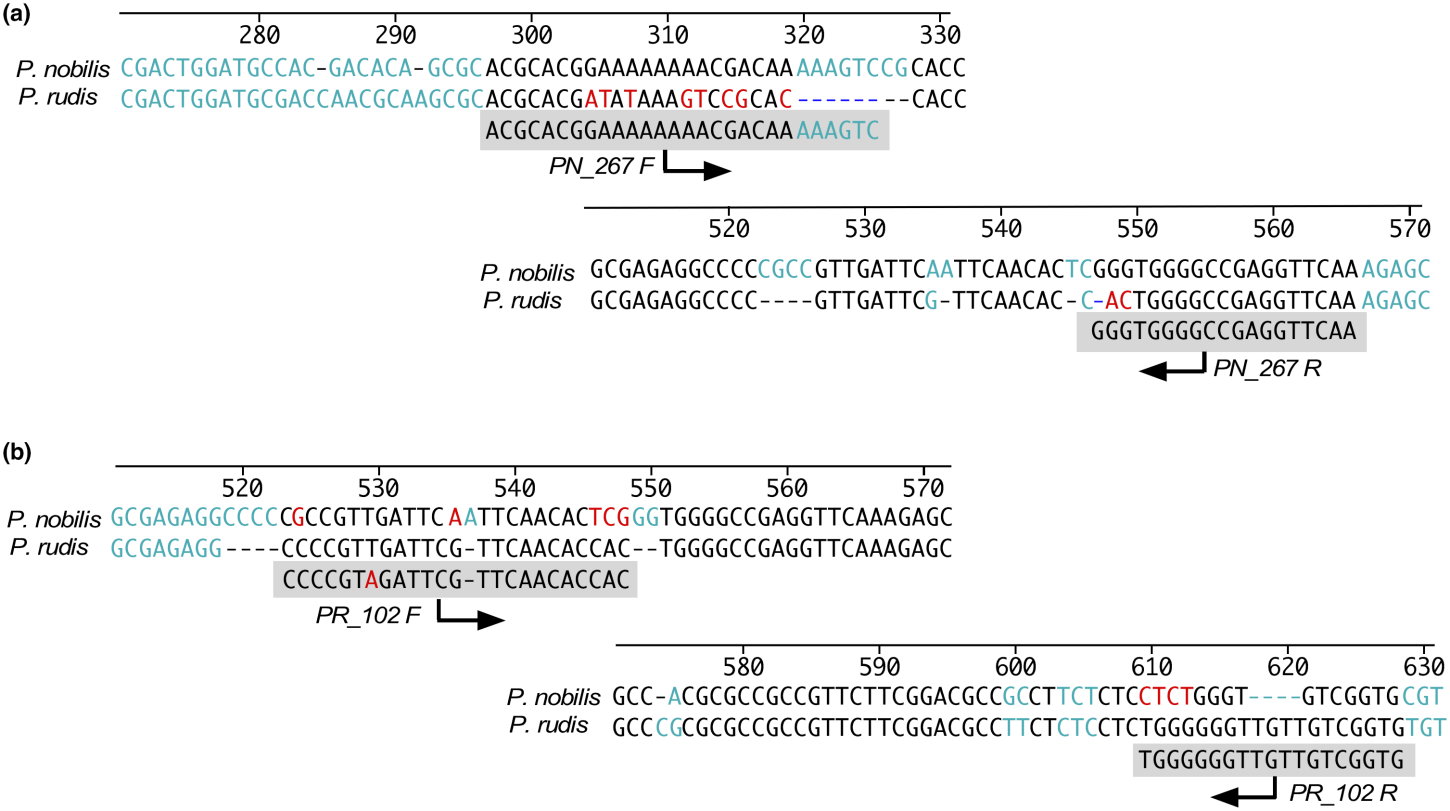
Primer positions in the ITS region for specific PCR amplifications: (a) Forward and reverse primers designed for the identification of *P. nobilis*; (b) Forward and reverse primers designed for the identification of *P. rudis*.

To lead it, we have designed species‐specific primers for each of the two species of the genus *Pinna* (Figure 1a,b). The specific primer pair for *P. nobilis* PCR amplification was PN_267 F (5′‐ACGCACGGAAAAAAAACGACAAAAAGTC‐3′), PN_267 R (5′‐TTGAACCTCGGCCCCACCC‐3′) while for *P. rudis* PCR amplification was PR_102 F (5′‐CCCCGTAGATTCGTTCAACACCAC‐3′), PN_102 R (5′‐CACCGACAACAACCCCCCA‐3′).

**FIGURE 2:**
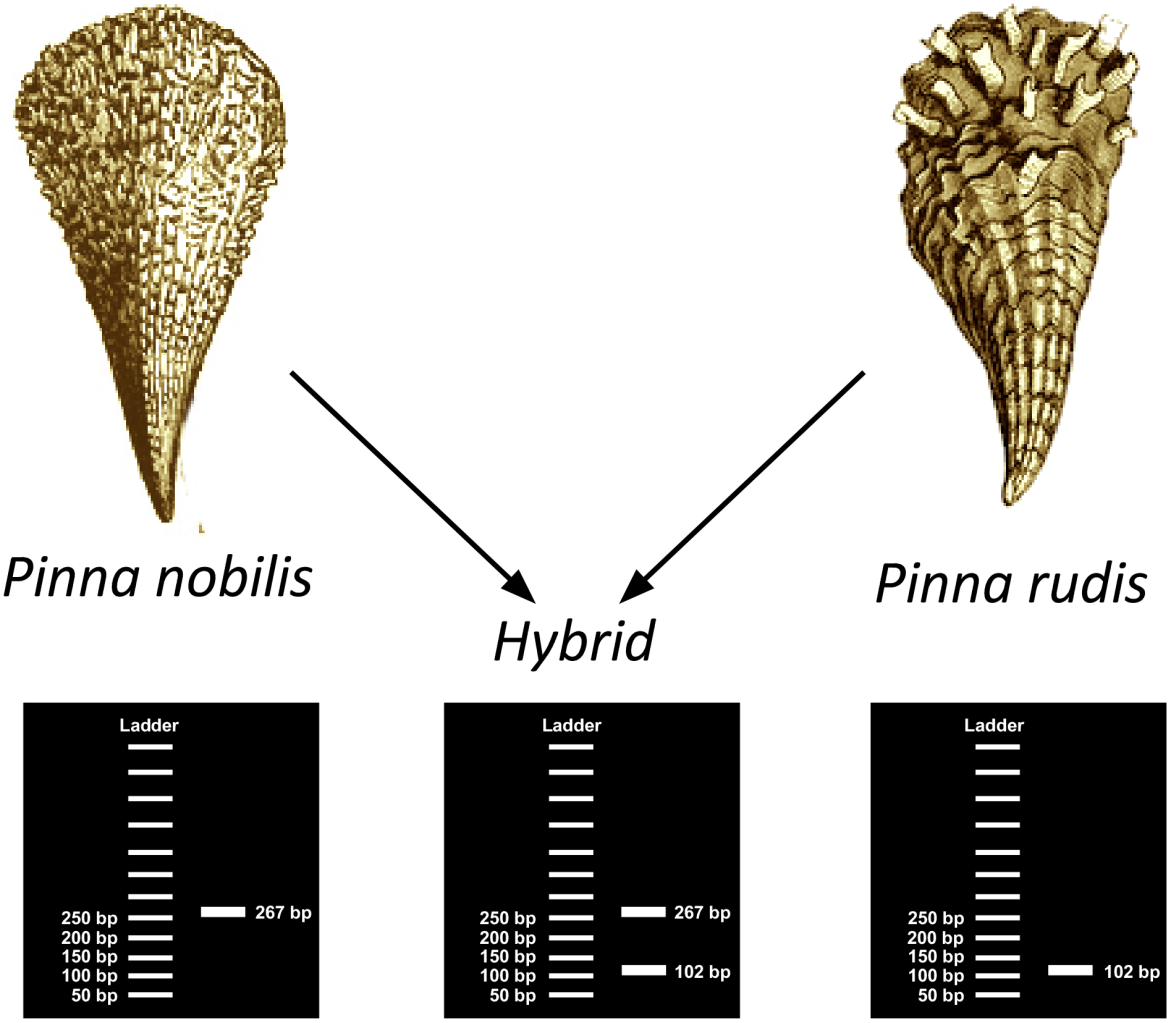
Overview of the developed PCR multiplex method for specific amplification and identification. This method targets *P. nobilis*, *P. rudis*, and their hybrids.

These primers have been designed in such a way that, after multiplex PCR amplification and subsequent visualization of the amplified products on an agarose gel, the different species can be differentiated based on the size of the specific amplified DNA fragments. In presence of a hybrid individual, two amplicons, one for each parental species, should be observed (Figure 2).

The multiplex PCR reaction using DNA was carried out in a total volume of 20 μL (microlitres) containing: 10 μL (1.25 units) of KAPATaq DNA polymerase (Merck), 0.3 μL of each primer PN_267F and PN_267R (20 μM) (specific for *P. nobilis*) and 0.4 μL of each primer PR_102F and PR_102R (20 μM) (specific for *P. rudis*), 1 μL of template DNA from the sample (∼30–50 ng) and sterile distilled water until the total volume of 20 μL.

The PCR amplifications were performed with KapaTaq DNA polymerase under the following conditions: an initial denaturation step at 95°C for 2 min, followed by 35 cycles of denaturation at 95°C for 30 s, annealing at 62°C for 10 s and elongation at 72°C for 20 s.

The PCR products were loaded onto high‐resolution agarose gels containing Ultrapure Agarose (Invitrogen) with a minimum concentration of 2.5% and subsequently stained using GelRed (Biotium). Electrophoresis was conducted in 1x TBE buffer, and DNA fragments were visualized under ultraviolet light. The size of DNA fragments was determined using the standard 50 bp DNA Ladder (Norgen). This method facilitates the direct identification of each *Pinna* species through the observation of distinct PCR band sizes in agarose gel, eliminating the need for additional sequencing.

Then, we analysed samples collected from juveniles captured in collectors and samples of adults exhibiting morphological characteristics of both species. This analysis was conducted using the described specified method, in addition to the method reliant on species identification through mitochondrial DNA analysis (Catanese, Coupé, & Bunet, 2022; Catanese, Tena‐Medialdea, et al., 2022).

## 3 RESULTS

The different sequences of ITS regions were sent to GenBank and used for sequence alignment and phylogenetic tree. We characterized the ITS region of following bivalve species: *P. nobilis* (access. n. LC794141), *P. rudis* (LC794142); *Pinctada* sp (LC814847‐LC814850); *Isognomon* sp. (LC814845); *Malleus* sp. (LC814846) and we confirmed the already described sequences of *Spondylus gaederopus* (LC814851‐LC814852) and *Atrina pectinata* (LC814844).

The obtained sequences containing ITS regions varied in length from 1046 to 1377 bp approximately. The multiple sequence alignment revealed the presence of a more conserved region identified as 5.8S rRNA within the sequences from the studied species.

Considering only the comparison between the sequences obtained from *P. nobilis* and *P. rudis*, the alignment between them highlighted 107 polymorphic sites, 67 of which were indels and 40 nucleotide substitutions. No differences were observed among specimens of *Pinna* species from different geographic sampled areas sampled in the Mediterranean Sea.

The ITS regions of all these organisms proved to have similar base composition to those typical of the vast majority of other marine organisms with 51%–59% GC content (Chow et al., 2009).

### 3.1 Genetic distance and phylogenetic analysis

The genetic distance among the analysed sequences varied from 0.457 in the comparison between *Malleus* sp. and *Isognomon* sp. to 0.847 in the comparison between *S. gaederopus* and *Haliotis iris* (Table S1).

**FIGURE 3:**
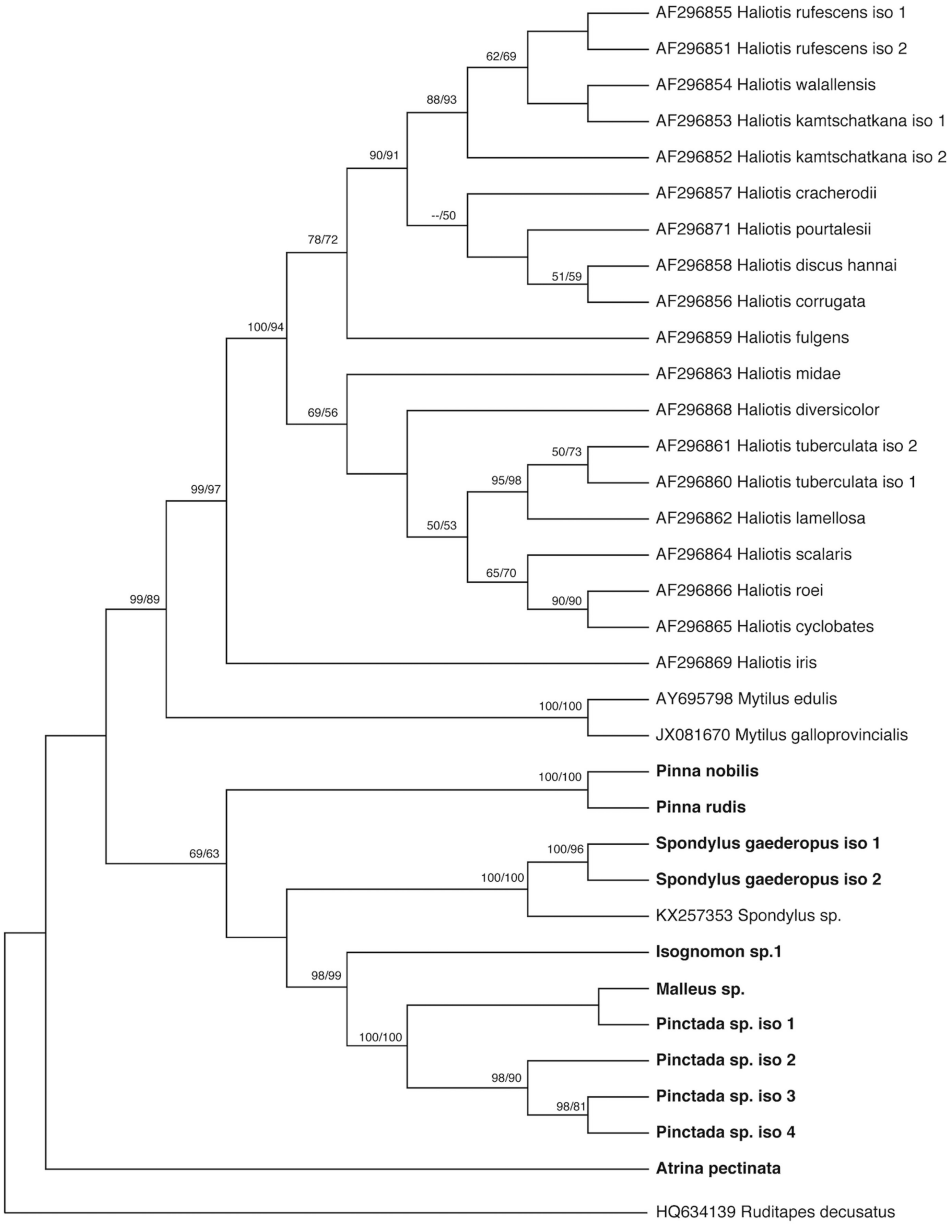
Phylogenetic relationships based on ITS sequences from various bivalve families. Bootstrap values from neighbour‐joining and maximum likelihood analyses greater than 50% are shown below the nodes, respectively.

For phylogenetic analysis, the Akaike Information Criterion was applied using JModeltest software, indicating the general time reversible (GTR) as the best‐fit model of DNA sequence evolution, which was applied to build the ML tree. A neighbour‐joining, was also inferred using Tamura‐Nei genetic distances from the ITS sequences. The Phylogenetic trees resulting from the NJ and ML analyses were almost congruent with each other (Figure 3).

The sequences of *Pinna* species were grouped in the same cluster, with high bootstrap (100%) and clearly separated from all other sequences.

### 3.2 Identification of Pinna sp. and hybrids

Analysing the alignment of ITS sequences between *P. nobilis* and *P. rudis*, to search specific primers for identification, the positions 320–326 and 546–549 were chosen as optimal to design the *P. nobilis*‐specific forward (PN_267 F) and reverse primers (PN_267 R) because the sequence permitted the detection of the nucleotide differences at the critical 3‐end between the two species (Figure 1a). The amplicon expected was 267 bp in length. This *P. nobilis*‐specific sequence stretch was completely conserved in all the sequences obtained in this work. Moreover, differences were observed in the comparison of the sequence of the primer regions in the other bivalves used in this study.

Similarly, the positions 545–547 and 609–612 of alignment were chosen as optimal for *P. rudis*‐specific forward (PR_102 F) and reverse primer (PR_102 R) which also showed nucleotide differences at the critical 3‐end (Figure 1b). The expected length of this specific amplicon was 102 bp. The other bivalves also showed greater differences in the sequence of the region of primers.

**FIGURE 4:**
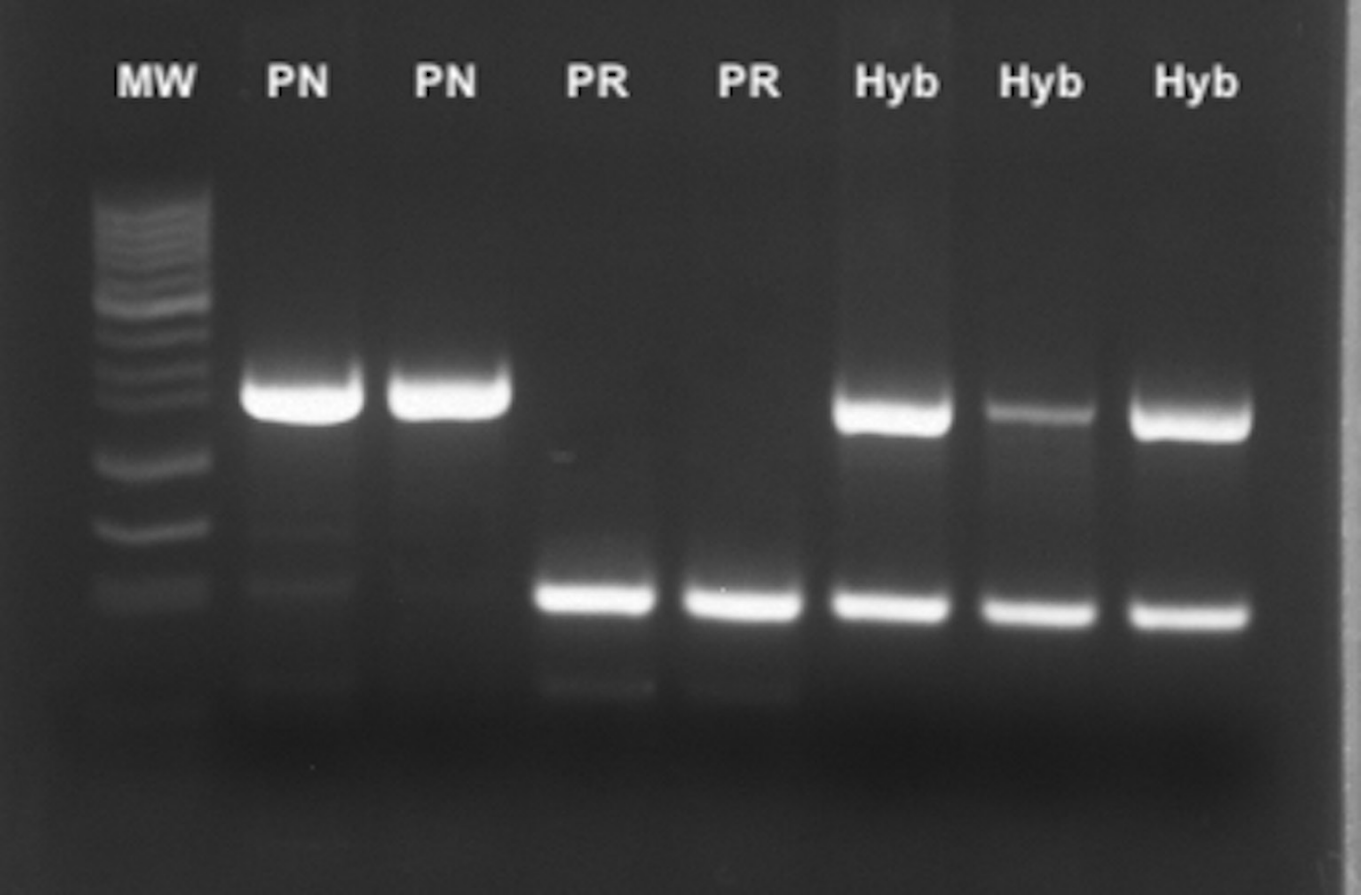
Electrophoresis in Agarose gel of multiplex‐PCR products from DNA of adult *Pinna* sp. individuals. Band sizes of the 50 bp DNA ladder (MW) are indicated on the left. PCR products are displayed as follows: Lanes 1–2: *P. nobilis* samples (PN); Lanes 3–4: *P. rudis* samples (PR); Lanes 5–7: Hybrid samples (Hyb).

For the multiplex PCR, in addition to *P. nobilis* and *P. rudis* we tested different bivalve and three putative hybrid samples from specimens which showed indistinguishable morphological characteristics between the two species. No amplification was obtained for the other bivalve species (data not shown). In contrast, samples from *P. nobilis* and *P. rudis* exhibited amplification of the species‐specific fragment. Additionally, amplification of both PCR fragments was observed in the three putative hybrid samples, confirming the effectiveness of the developed method (Figure 4).

The same samples indicated as putative hybrids were previously identified using the mitochondrial analysis method described by Catanese, Tena‐Medialdea, et al. (2022) as *P. nobilis*, which would confirm uniparental mitochondrial transmission in these species although a situation of hybridization or gene introgression between the two species is taking place.

## 4 DISCUSSION

Natural hybridization plays a fundamental role in evolution due to its ability to influence genetic diversity, adaptation and speciation processes. This phenomenon involves genetic interaction between individuals of different species. At least 25% of plant species and 10% of animal species, mostly the youngest species, are involved in hybridization and potential introgression with other species (Mallet, 2005). Hybridization between marine species has been observed across various contexts. Examples include hybridization between the grey seal (*Halichoerus grypus*) and the common seal (*Phoca vitulina*) in the North Atlantic (Helyar et al., 2011), the bottlenose dolphin (*Tursiops truncatus*) and Risso's dolphin (*Grampus griseus*) in the Gulf of Mexico (Mazzoil et al., 2018), the American horseshoe crab (*Limulus polyphemus*) and Japanese horseshoe crab (*Tachypleus tridentatus*) (Shuster Jr et al., 2004), and different species of angelfish (*Pterophyllum scalare* and *Pterophyllum altum*) in South America (Le Bail & Keith, 2007).

In general, interbreeding between evolutionarily distinct lineages is termed “hybridization,” whereas “introgression” denotes gene flow between species resulting from this hybridization (Pfennig et al., 2016). Natural hybridization between species can introduce new alleles into populations, thus increasing genetic variability, and generating genetic combinations that confer adaptive advantages (Arnold, 1997). In fact, hybrid individuals may exhibit different phenotypes or combinations of genetic characteristics from the parental species, allowing them to adapt to environmental changes and new selective pressures. Hybridization can contribute to the generation of new hybrid species with unique characteristics.

However, even if the presence of hybrids can lead to an increase in genetic variability, genetic introgression can also compromise the survival of native populations. Although hybrid species could represent a resource for adapting to environmental changes by inheriting beneficial characteristics from their parents and favouring their survival in new environmental conditions, hybridization can threaten the genetic purity of the original species, especially if the parental species are at risk of extinction or are local endemics. If hybrid species show greater reproductive success or adaptation to the local environment, they could gradually displace parental species, leading to changes in the composition of marine communities (Todesco et al., 2016). Therefore, the fertility of hybrid species is an important aspect to consider in relation to their ecology and conservation. It can vary greatly depending on the species involved and the type of hybridization. Some hybrid species may be fertile and able to reproduce, while others may be sterile or have reduced fertility. Fertility is crucial to determining whether they can form a stable population in the long term. Indeed, they may contribute to a new gene pool that could evolve independently of the parental species.

In the case of *Pinna* hybrids, it is not known whether the first generation of offspring resulting from the crossbreeding of the two distinct parental lines (F1 generation) are fertile or infertile. If they were fertile and reproductively successful, they may require specific management in conservation.

Some authors emphasized the need for clear guidelines in conservation efforts to address hybridization issues effectively. Allendorf et al. (2001) proposed strategies for managing hybridization, including assessing the genetic and ecological impacts of hybrids and implementing appropriate management actions to conserve species integrity and diversity.

The study of hybridization events and the formation of sterile and non‐sterile hybrids can also provide information on the processes of speciation and diversification, the mechanisms that promote reproductive isolation between species and the different adaptability or diverse or fluctuating ecological niches. Furthermore, understanding the dynamics and consequences of hybridization is essential for biodiversity management and conservation.

However, even if the presence of hybrids can lead to an increase in genetic variability, it must be considered that hybrids could also indicate a problem since genetic introgression can also compromise the survival of native populations.

Hybrids often possess a broader range of ecological traits than their parent species. This broader ecological niche may include differences in habitat use and feeding behaviours. Greater ecological flexibility can reduce exposure to specific pathogens or minimize disease transmission within populations, thus contributing to overall resilience.

Considering that until now *P. rudis* has not shown massive mortality events, probably as a consequence of its greater adaptability to environmental changes and/or the presence of pathogens, hybrid individuals could also possess a greater adaptive potential to cope with emerging pathogens or changing environmental conditions. Rapid evolution through hybridization can lead to the development of new genetic combinations that confer resistance to prevalent pathogens.

Genome of the *P. nobilis* × *P. rudis* hybrids seems to be naturally resistant to the parasite (Vázquez‐Luis et al., 2021), suggesting that their innate immunity could be efficient enough to prevent from infection development and spreading.

Recently, RNA‐Seq comparative study revealed molecular effectors linked to the resistance of *P. nobilis* to *H. pinnae* parasite (Salis et al., 2022), while a preliminary research suggests a role of fast‐evolving immune gene TLR‐7 (toll‐like receptor) in *P. nobilis* resistance to *H. pinnae*, with possible TLR‐6 and TLR‐4 influence (Coupé et al., 2023). Although further studies are needed to clarify the role of TLRs in disease resistance, the presence of TLR genes from *P. rudis* in the genome of *P. nobilis* individuals resistant to *H. pinnae*, but not in those susceptible to the pathogen, suggests that resistance genes may have arisen through different ancestral evolutionary processes or have been introduced through adaptive hybridization over several generations.

However, our method was primarily designed to reliably identify first‐generation hybrids. Detecting individuals from older hybridization events, in the case of F1 hybrids are fertile, can be challenging. As F2 and subsequent generation hybrids may rapidly diverge both phenotypically and genotypically from F1 hybrids, accurate identification of these later‐generation hybrids could require the molecular analysis of multiple loci.

Anyway, from a conservation perspective, it is crucial to possess tools capable of detecting hybrids at the molecular level for several important reasons, particularly to ascertain the presence and scope of hybridization within natural populations. This information is vital for delineating conservation units and establishing conservation priorities, especially in scenarios where hybridization may pose risks or confer an advantage to endemic or endangered species.

Natural hybridization in molluscs is an intriguing phenomenon where individuals of different species can reproduce and produce viable offspring. Closely related mussel species engage in documented natural hybridization, especially in coastal areas with specific marine currents. In the specific case of mussels, it has been observed that they can hybridize in areas where their distributions overlap (Simonsen, 2001). For example, hybridization between the mussel species *M. edulis* and *M. galloprovincialis* and evidence of intragenic recombination in a genetic marker was found by Rawson et al. (1996) Furthermore, several researchers have investigated the genetic structure and distribution of hybrid mussel populations in various geographical regions (Coghlan & Gosling, 2007; Gosling et al., 2008; Inoue et al., 1997; Kartavtsev et al., 2014). In *Pinna* species, individuals featuring intermingled shell morphology and mantle coloration, with both *P. nobilis* and *P. rudis* traits have been observed. They were identified as putative hybrids by sequencing analysis detecting 9 diagnostic nucleotides of the 28S nuclear gene (Vázquez‐Luis et al., 2021). In our work, we used the same individuals for testing the developed method.

Several studies have incorporated phylogenetic analysis of the spacer regions of nuclear ribosomal genes. The ITS regions are particularly valuable in phylogenetic and taxonomic studies for inferring evolutionary relationships between species, owing to the conservation and co‐evolution of ribosomal unit sequences (Baldwin, 1992).

The ITS regions have emerged as a valuable tool for assessing plant, fungal, and algal lineages across various taxonomic levels due to their ease of amplification through PCR and sequenceability using conservative primers, proposing them as a potential barcode and for phylogenetic studies (Letsiou et al., 2024; Schoch et al., 2012; Stern et al., 2012; Yao et al., 2010). They provide high information content due to their variability between species and conservation within individuals of the same species, attributed to concerted evolution associated with the entire ribosomal unit. Concerted evolution refers to coordinated or simultaneous changes over time in DNA sequences encoding ribosomal subunits (rRNA) within an organism, ensuring the functional integrity and efficiency of the ribosomal system (Hillis et al., 1991).

The reconstruction of the phylogenetic history of some mollusc species has been attempted previously by different authors, using different gene loci and other aspects of DNA. In this study, the phylogenetic analyses were conducted on a partial scale rather than in‐depth, focusing solely on confirming significant molecular differences in ITS regions between species of taxonomically close families. Our goal was to ensure accurate primer design specific to *Pinna* species. Although the uniformity in sequence across specimens of *Pinna* species from various Mediterranean regions supports our results, further analysis of additional samples is needed to validate the observed low variability.

Finally, the choice between using a species‐specific PCR or sequencing a DNA fragment to detect hybrids between species is essential considering the practical and technical benefits of each approach. Species‐specific PCR offers a significant advantage in terms of sensitivity and specificity as well as in time and cost (Edwards & Gibbs, 1994) It allows highly accurate detection of sequences of interest, minimizing the possibility of cross‐amplification or false positives (Edwards & Gibbs, 1994). This precision is essential when searching for hybrids that may have genetic sequences similar but not identical to those of their parental species. The detection of hybrids between species can be indicated by successful amplification of the PCR product using primers designed for each parental species (Gross et al., 1996). In contrast, DNA sequencing can introduce ambiguities in interpretation when “double peak” patterns within DNA chromatograms are observed at nucleotide positions where parental sequences differ. The double peaks may complicate accurate identification of hybrids and could require additional bioinformatic analyses to confirm the presence of heterozygosity and hybridization.

Furthermore, species‐specific PCR requires less genetic material to carry out amplification than DNA sequencing, which is especially useful when working with limited or unpurified samples. The costs associated with species‐specific PCR are generally lower in terms of reagents and analysis time compared to DNA sequencing, making it a more accessible and practical option in many research contexts.

Ease of implementation is also a point in favour of species‐specific PCR. This technique is well established in molecular biology laboratories and the necessary protocols and reagents are widely available. This facilitates its adoption and application in studies to detect hybrids between species, allowing researchers to obtain reliable results quickly and effectively. Moreover, this technique is highly recommended for both adult and juvenile individuals. It can be particularly effective when applied to fresh or frozen eDNA samples, eliminating the need for direct manipulation of individuals during sampling, similar to the mitochondrial assay used for *Pinna* identification.

In conclusion, species‐specific PCR emerges as a preferred strategy over DNA sequencing when seeking to detect hybrids between species. It offers sensitivity, specificity and efficiency, allowing clear and precise identification of genetic crosses between different species. This robust and practical technique is essential to advance our understanding of genetic diversity and evolution in contexts of interspecies hybridization.

The ongoing mortality event affecting *Pinna nobilis* in the Mediterranean Sea remains a critical concern for conservation efforts. Efforts to safeguard surviving populations of *P. nobilis* involve a multi‐pronged approach, including monitoring, captive breeding and employing collectors for juvenile recruitment. Captured juveniles are screened for pathogens and used in repopulation initiatives, in sites with specific local abiotic conditions such as extreme salinity or low temperature.

Hybridization events between *P. nobilis* and *P. rudis* could help conservation efforts due to their possible resilience to infection. Molecular techniques utilizing ITS regions enable the precise identification of hybrids and parental species, providing valuable insights into genetic interactions and potential introgression. Species‐specific PCR assays have proven effective in distinguishing hybrids, offering a practical and cost‐effective means of detecting genetic crosses.

The study underscores the significant implications of hybrids and the importance of using molecular tools for their identification. This is critical for effective biodiversity management and conservation strategies, ultimately ensuring the preservation of populations and deepening our understanding of the evolutionary consequences arising from genetic interactions between different species. Functional genomics research could reveal how hybridization affects physiology, behaviour, and ecological interactions, informing conservation plans. Additionally, studying the genetic resilience of *Pinna* species and their hybrids to climate change would turn out crucial for understanding adaptive capacities to rising sea temperatures and ocean acidification.

In conclusion, ongoing collaborative efforts involving scientists, environmentalists, and government organizations are essential for implementing effective monitoring, conservation, and repopulation strategies to safeguard *P. nobilis* populations against mortality events and emerging hybridization dynamics.

## AUTHOR CONTRIBUTIONS

**Gaetano Catanese:** Conceptualization (lead); formal analysis (lead); funding acquisition (supporting); investigation (equal); methodology (lead); writing – original draft (lead); writing – review and editing (equal). **Maite Vázquez‐Luis:** Funding acquisition (supporting); investigation (equal); project administration (lead); resources (equal); writing – review and editing (equal). **Salvatore Giacobbe:** Investigation (equal); resources (equal); writing – review and editing (equal). **José Rafael García‐March:** Investigation (equal); project administration (equal); resources (equal); writing – review and editing (equal). **Maria Zotou:** Investigation (equal); resources (equal); writing – review and editing (equal). **Prado Patricia:** Investigation (equal); resources (equal); writing – review and editing (equal). **Orestis Papadakis:** Investigation (equal); resources (equal); writing – review and editing (equal). **José Tena‐Medialdea:** Investigation (equal); resources (equal); writing – review and editing (equal). **Stelios Katsanevakis:** Investigation (equal); resources (equal); writing – review and editing (equal). **Amalia Grau:** Funding acquisition (lead); investigation (equal); writing – review and editing (equal).

## CONFLICT OF INTEREST STATEMENT

The authors declare no conflict of interest.

## Supporting information


Data S1.

## Data Availability

All experimental and unique haplotype data are available in the manuscript and deposited to NCBI Nucleotide Database (GenBank accession codes LC794141, LC794142 and from LC814844 to LC814852).
